# Loss of Keratin 8 Phosphorylation Leads to Increased Tumor Progression and Correlates with Clinico-Pathological Parameters of OSCC Patients

**DOI:** 10.1371/journal.pone.0027767

**Published:** 2011-11-17

**Authors:** Hunain Alam, Prakash Gangadaran, Amruta V. Bhate, Devendra A. Chaukar, Sharada S. Sawant, Richa Tiwari, Jyoti Bobade, Sadhana Kannan, Anil K. D'cruz, Shubhada Kane, Milind M. Vaidya

**Affiliations:** 1 Cancer Research Institute (CRI), Advanced Centre for Treatment, Research and Education in Cancer (ACTREC), Tata Memorial Centre (TMC), Kharghar, Navi Mumbai, India; 2 Surgical Oncology, Head and Neck Unit, Tata Memorial Hospital (TMH), Parel, Mumbai, India; 3 Epidemiology and Clinical Trials Unit, Clinical Research Centre (CRC), Advanced Centre for Treatment, Research and Education in Cancer (ACTREC), Tata Memorial Centre (TMC), Kharghar, Navi Mumbai, India; 4 Department of Pathology, Tata Memorial Hospital (TMH), Parel, Mumbai, India; Sanford Burnham Medical Research Institute, United States of America

## Abstract

**Background:**

Keratins are cytoplasmic intermediate filament proteins expressed in tissue specific and differentiation dependent manner. Keratins 8 and 18 (K8 and K18) are predominantly expressed in simple epithelial tissues and perform both mechanical and regulatory functions. Aberrant expression of K8 and K18 is associated with neoplastic progression, invasion and poor prognosis in human oral squamous cell carcinomas (OSCCs). K8 and K18 undergo several post-translational modifications including phosphorylation, which are known to regulate their functions in various cellular processes. Although, K8 and K18 phosphorylation is known to regulate cell cycle, cell growth and apoptosis, its significance in cell migration and/or neoplastic progression is largely unknown. In the present study we have investigated the role of K8 phosphorylation in cell migration and/or neoplastic progression in OSCC.

**Methodology and Principal Findings:**

To understand the role of K8 phosphorylation in neoplastic progression of OSCC, shRNA-resistant K8 phospho-mutants of Ser73 and Ser431 were overexpressed in K8-knockdown human AW13516 cells (derived from SCC of tongue; generated previously). Wound healing assays and tumor growth in NOD-SCID mice were performed to analyze the cell motility and tumorigenicity respectively in overexpressed clones. The overexpressed K8 phospho-mutants clones showed significant increase in cell migration and tumorigenicity as compared with K8 wild type clones. Furthermore, loss of K8 Ser73 and Ser431 phosphorylation was also observed in human OSCC tissues analyzed by immunohistochemistry, where their dephosphorylation significantly correlated with size, lymph node metastasis and stage of the tumor.

**Conclusion and Significance:**

Our results provide first evidence of a potential role of K8 phosphorylation in cell migration and/or tumorigenicity in OSCC. Moreover, correlation studies of K8 dephosphorylation with clinico-pathological parameters of OSCC patients also suggest its possible use in prognostication of human OSCC.

## Introduction

Keratins (K) are largest subgroup of intermediate filament (IF) proteins preferentially expressed in epithelial tissues [1], [2]. They are subdivided into type I acidic (K9–K28) and type II basic (K1–K8 and K71–K74) keratins [2], [3] on the basis of their biochemical properties such as molecular weight and isoelectric point [4]. They are obligatory heteropolymers and are assembled in 1∶1 molar ratio, consisting of one type I and one type II keratins [1], [2], [5]. Epithelial tissues express different pairs of keratins depending on the cell type e.g. all stratified squamous epithelia express K5 and K14 [6] while K8 and K18 are seen in all simple epithelia [7].

K8 and K18 is the prototype keratin pair of simple epithelia and are predominantly expressed in epithelial components of glandular tissues, including the pancreas and intestine, with other keratins such as K7, K19 and K20 [8]. Apart from their known protective mechanical functions, K8 and K18 also execute various regulatory functions, which include modulation of protein localization, protein targeting/trafficking and protein synthesis [7], [9].

K8 and K18 expression is not observed in stratified adult epithelial tissues. However, they are often aberrantly expressed in carcinomas including oral SCC and their expression is correlated with invasion and poor prognosis [2], [10], [11]. Aberrant overexpression of K8 and K18 in SCC cell lines has been shown to promote tumorigenicity and cell migration. Previous data of our laboratory has shown that K8 overexpression leads to neoplastic transformation and increased invasive and metastatic potential in FBM cell line [12]. These observations were further supported by a transgenic study [13]. Recently, we have shown that K8 knockdown leads to reduction in cell migration and tumorigenicity accompanied with downregulation of β4-integrin-mediated signalling in OSCC cells [14].

All IF proteins, including keratins, consist of a conserved central coiled-coil α-helical- “rod” domain and flanking non–α-helical NH_2_-terminal “head” and COOH-terminal “tail” domains [5], [15]. Their post translational modifications including phosphorylation reside in flanking head and tail domains [16]. Keratin phosphorylation is dynamic process which affects the organization of filaments, either by increasing the exchange between the soluble and the cytoskeletal fraction or by regulating the binding sites of associated proteins e.g. 14-3-3 [16], [17]. It is also involved in the regulation of many protein functions which include protection against stress, signaling, apoptosis and cell compartment-specific roles [16], [17]. Most of the phosphorylation sites identified so far involve distinct serine (Ser) residues of keratins [16]. Several protein kinases, such as PKC, p38, ERK, cAMP and JNK are known to phosphorylate keratins [16], [17], [18]. Prior studies show that exposure of cells or tissues to phosphatase inhibitors causes dramatic hyperphosphorylation of K8 and K18 [19], [20], [21], [22]. Recently, specific phosphatases such as phosphatase of regenerating liver-3 (PRL-3) and protein phosphatase-2A (PP2A) have been identified which dephosphorylate K8 at specific residue [23], [24]. Hyperphosphorylation of K8 and K18 is observed under conditions of tissue injury, stress and apoptosis [17], [25]. Ser23, Ser73 and Ser431 are the three phosphorylation sites of K8 [16]. Phosphorylation of K8 at residue Ser431 observed in response to epidermal growth factor (EGF) stimulation [26]. Ser73 is phosphorylated in response to various types of cellular stresses [18]. A recent report suggests that K8 Ser73 and Ser431 dephosphorylation is associated with cell migration and metastasis in colorectal cancer [23]. However, the significance of K8 phosphorylation in neoplastic progression of SCC is not yet studied.

In the present study we have shown that overexpression of shRNA-resistant K8 phospho-mutants in K8-knockdown OSCC cells demonstrate increase in cell motility and tumorigenicity compared with K8-wild type clone. Furthermore, K8 dephosphorylation was also observed in human OSCC samples which significantly correlated with size, lymph node metastasis and stage of the tumor.

## Results

### Generation of shRNA-resistant K8-phospho-mutants and K8 wild type constructs

**Figure 1:**
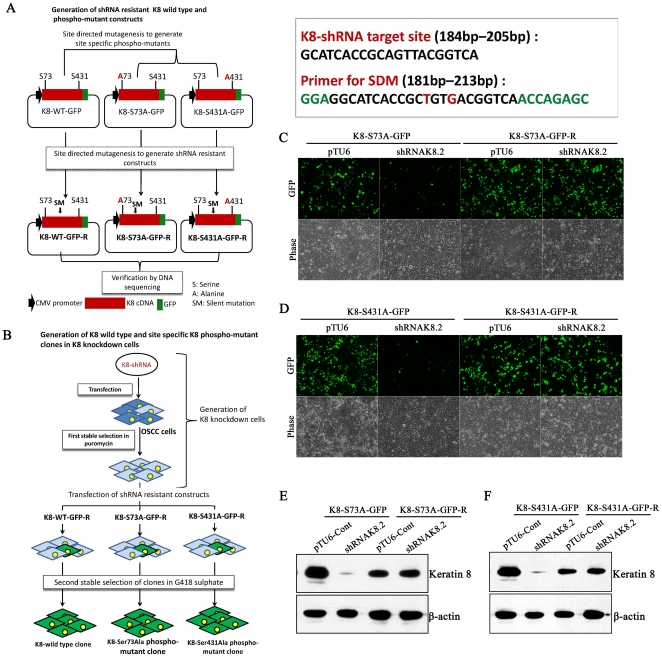
Generation of RNAi resistant K8 phospho-mutants constructs. (**A**) Strategy for generation of shRNA resistant K8 wild type and K8 phospho-mutants, Ser73Ala and Ser431Ala constructs. (**B**) Strategy for generation of K8 wild type and K8 phospho-mutants, Ser73Ala and Ser431Ala stable clones in K8-knockdown AW13516 cells. (**C and D**) Mutated RNAi resistant GFP-tagged K8 phospho-mutants, K8-S73A-GFP-R and K8-S431A-GFP-R were validated by cotransfecting them with shRNAK8.2 or vector pTU6-PURO in HEK293 cells. shRNA-sensitive GFP-tagged phospho-mutants (K8-S73A-GFP and K8-S431A-GFP) were also transfected separately in HEK293 cells and used as controls. Representative images of GFP expression 60 hrs of post-transfection (combination of transfection is indicated in figure; Magnification 10X). (**E and F**) Western blot analysis of proteins extracted from cells transfected with shRNA-resistant (K8-S73A-GFP-R and K8-S431A-GFP-R) and their respective shRNA-sensitive constructs (K8-S73-GFP and K8-S431-GFP) using K8 antibody (order of samples is indicated in figure). β-actin was used as loading control.

In order to express K8 phpspho-mutants and K8-wild type in K8 knockdown AW13516 cells, shRNAK8.2 resistant GFP tagged K8 phospho-mutants and K8-wild type (described above) were generated by site directed mutagenesis. The resistance of resulted mutants against shRNAK8.2 was checked by cotransfecting them with shRNAK8.2 in HEK-293 cells followed by measurement of fluorescence intensity and western blot analysis (detail strategy of generation of these constructs has been described as flow chart in Figure 1A). The GFP tagged K8-Ser73Ala phospho-mutant (K8-S73A-GFP-R) and K8-Ser431Ala phospho-mutant (K8-S431A-GFP-R) constructs were found to be resistant to shRNAK8.2 (Figure 1C-F).

### Overexpression of K8-phospho-mutants in K8-knockdown AW13516 cells

**Figure 2:**
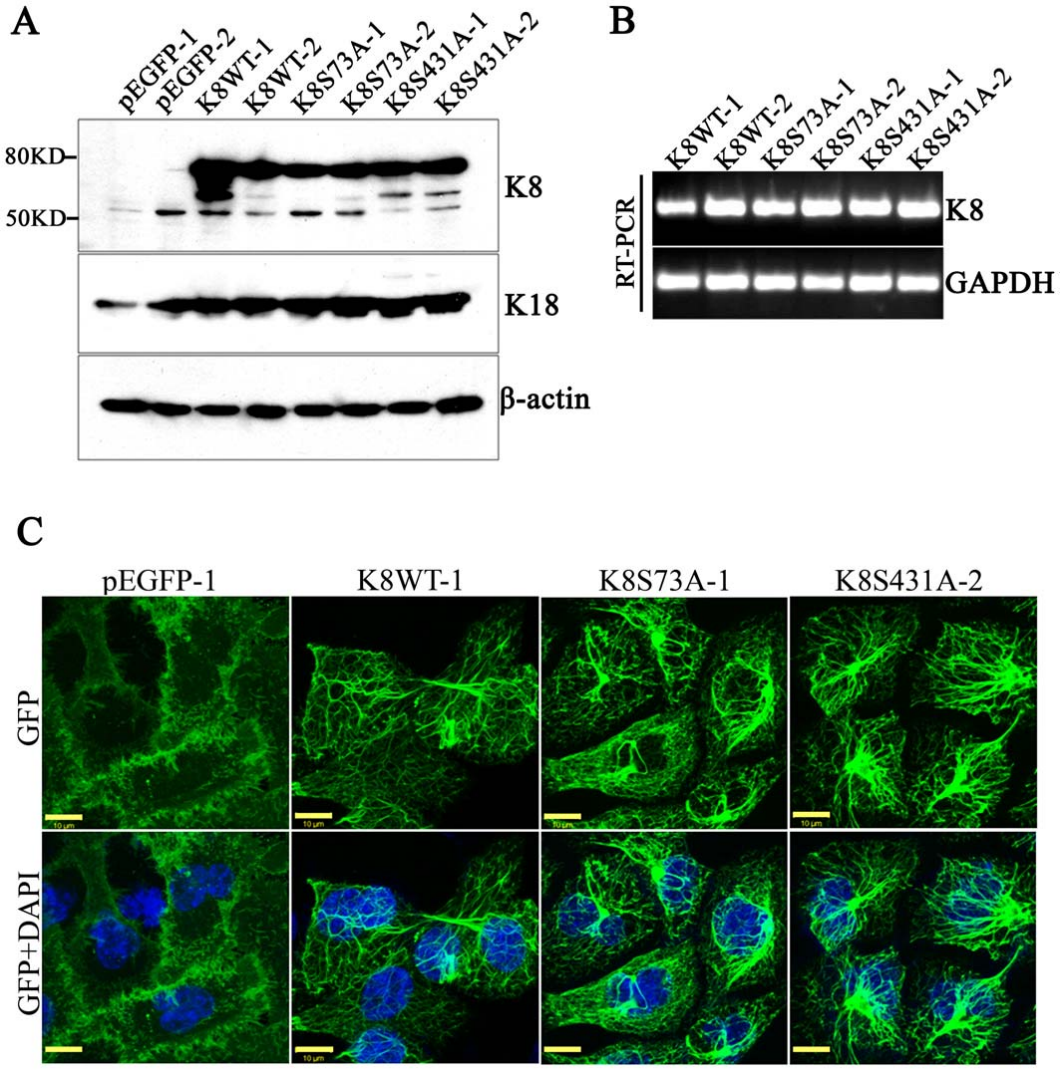
Stable overexpression of GFP-tagged K8 phospo-mutants in K8 knockdown AW13516 cells. (**A**) Western blot analysis of stable overexpressed K8 wild type (K8WT-1 and -2), K8-Ser73Ala phospho-mutant (K8S73A-1 and -2), K8-Ser431Ala phospho-mutant (K8S431A-1 and -2) and pEGFP (pEGFP-1 and -2) clones derived from K8 knockdown cells with antibodies to K8 and K18. β-actin was used as loading control. (**B**) RT-PCR analysis of K8 in stable K8 wild type and phospho-mutant clones. *GAPDH* was used as internal control. (**C**) Representative confocal images of filaments formed by GFP-tagged K8 in stable K8WT-1, KS73A-1 and K8S431A-2 clones. pEGFP-N3 empty vector transfected (pEGFP-1) clone was taken as control. Scale bar: 50 µm.

**Figure 3:**
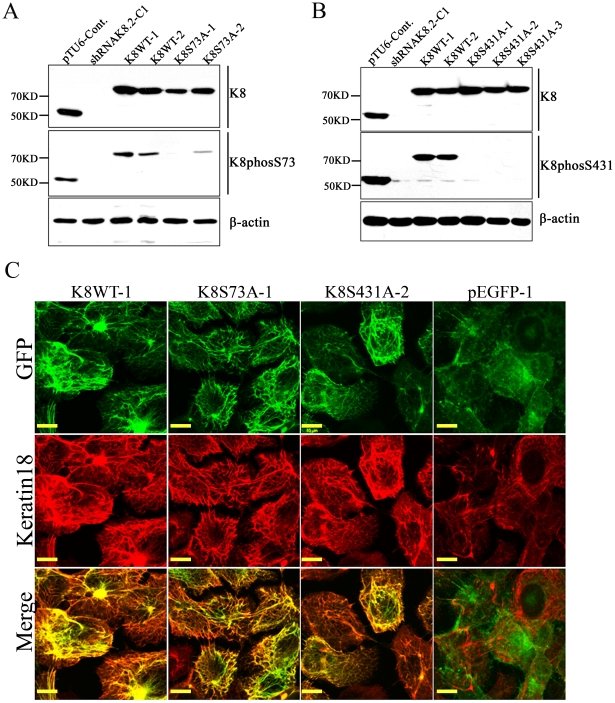
Analysis of K8 phosphorylation at Ser73 and Ser431 and their filament formation with K18 in stable K8 phospho-mutant clones. (**A and B**) Western blot analysis of stable overexpressed K8 wild type (K8WT-1 and -2), K8 Ser73Ala phospho-mutant (K8S73A-1 and -2) and K8 Ser431Ala phospho-mutant (K8S431A-1,-2 and -3) clones with antibodies against K8 or phospho-specific K8 Ser73 or Ser431. K8-knockdown (shRNAK8.2-C1) and vector control (pTU6-Cont) clone were taken as controls. β-actin was used to ensure equal loading. (**C**) Representative confocal micrographs of filaments formed by GFP-tagged K8 with endogenous K18 in K8 wild type (K8WT-1), K8Ser73Ala (K8S73A-1) and K8Ser431Ala (K8S431A-2) clones. Stably overexpressed empty vector clone (pEGFP-1) was taken as control. Scale bar: 50 µm.

In order to investigate the role of individual K8 phosphorylation (Ser73 and Ser431) during tumor progression in OSCC, shRNA-resistant K8-Ser73Ala and K8-Ser431Ala phospho-mutants were transfected in K8 deficient AW13516 C1-shRNAK8.2 cells. Stably over-expressed GFP tagged K8 wild type (K8WT-1and -2), K8-Ser73Ala mutant (K8S73A-1 and -2) and K8- Ser431Ala mutant (K8S431A-1 and -2) clones were selected in 1000 ug/ml G418-sulphate (detail strategy of generation of the K8-phospsho-mutant clones has been described as flow chart in Figure 1B). The over-expression of exogenous GFP-tagged K8 in stable clones (K8WT-1 and -2; K8S73A-1 and -2; K8S431A-1 and -2) was analyzed by western blot and RT-PCR analysis. All the clones showed considerable overexpression of exogenous K8 (Figure 2A, B). Western blot analysis using antibody specific to phosphorylated Ser73 or Ser431 of K8 confirmed the reduction of K8 phosphorylation on respective site in the phospho-mutant overexpressed clones (Figure 3A, B). Further, we also observed increase in K18 levels in these over-expressed clones by western blot analysis (Figure 2A). In addition, the filament formation by exogenously expressed GFP tagged K8 was analyzed by confocal microscopy. All the K8 wild type and phospho-mutant clones formed proper filaments with endogenous K18 (Figure 2B, 3C). There was no considerable difference in filament organization observed in the cells expressing K8 phospho-mutants compared with the cells expressing wild type K8. In addition, we have also observed considerable increase in K18 levels in both phospho-mutants and wild type stable clones (Figure 3A, B).

### Loss of K8 phosphorylation leads to increase in cell migration

**Figure 4:**
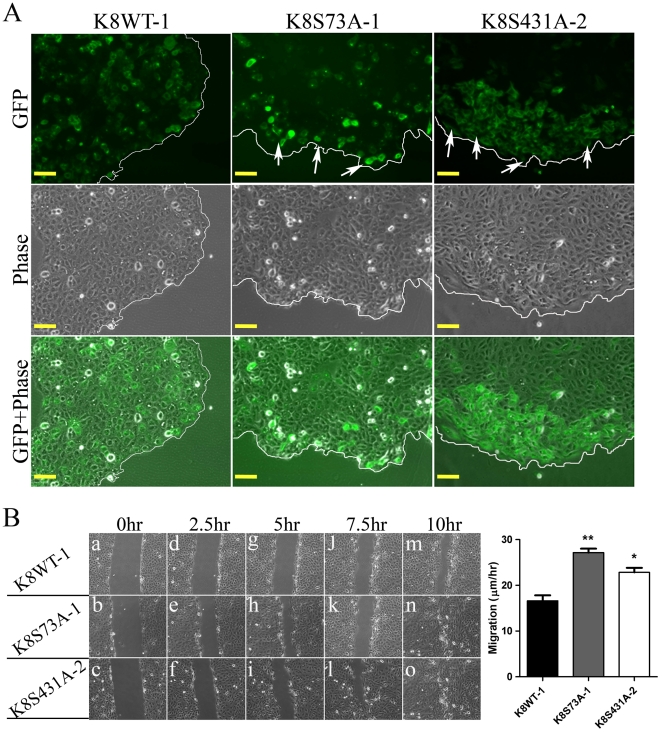
Effect of decreased K8 Ser 73 or Ser431 phosphorylation on migratory ability of OSCC cells. (**A**) Representative fluorescence and phase contrast micrographs of K8 wild type (K8WT-1) and K8 phospho-mutants (K8S73A-1 or K8S431A-2) cells that displayed GFP-tagged K8 analyzed by fluorescence microscopy. Note that the cells expressing high levels of K8-Ser73Ala or K8-Ser431Ala mutant appeared on the edges of the colony. Scale bar: 50 µm. (**B**) Scratch wound healing assays were performed on K8 wild type (K8WT-1), K8-Ser73Ala (K8S73A-1) and K8-Ser431Ala (K8S431A-2) clones. Phase contrast images (10X) of wound closure at 0 hr (panels a–c), 2.5 hr (panels d–f), 5 hr (panels g–i), 7.5 hr (panels j–l), and 10 hr (panels m–o) of the clones are shown in the figure. Scale bar: 100 µm. Migration rate was calculated by AxioVision software. The data shown is the average from three independent experiments with the mean and standard deviation. *P<0.05, ** *P*<0.01 (by student *t-*test).

Several prior studies demonstrated the role of keratin filaments in cell migration and wound healing [27]. During cell migration, keratin filaments undergo reorganization and their phosphorylation has been shown to regulate filament organization during various patho-physiological conditions [16], [17]. In order to determine the effect of loss of K8 phosphorylation on migratory behavior of the cells, first distribution of GFP-tagged exogenous K8 was analyzed by inverted fluorescence microscopy (Axio Vision 2000, Zeiss). The highly expressing GFP-tagged K8-Ser73Ala phospho-mutant and K8-Ser431Ala phospho-mutant cells were found to be on the edge of the colonies, while the GFP tagged K8 wild type expressing cells were uniformly distributed throughout the colonies (Figure 4A). These results suggested that loss of phosphorylation may lead to increase in migratory ability of the cells. Further, to confirm the effect of loss of K8 phosphorylation on cell migration, wound healing assay was performed. The stable K8-Ser73Ala and K8-Ser431Ala mutant clones demonstrated significant increase in cell migration compared to wild type clone (Figure 4B). These results together indicated that loss of K8 phosphorylation at Ser73 and Ser431 leads to increase in migratory ability of the OSCC derived cells.

### Loss of K8 phosphorylation resulted in increased tumorigenicity

**Figure 5:**
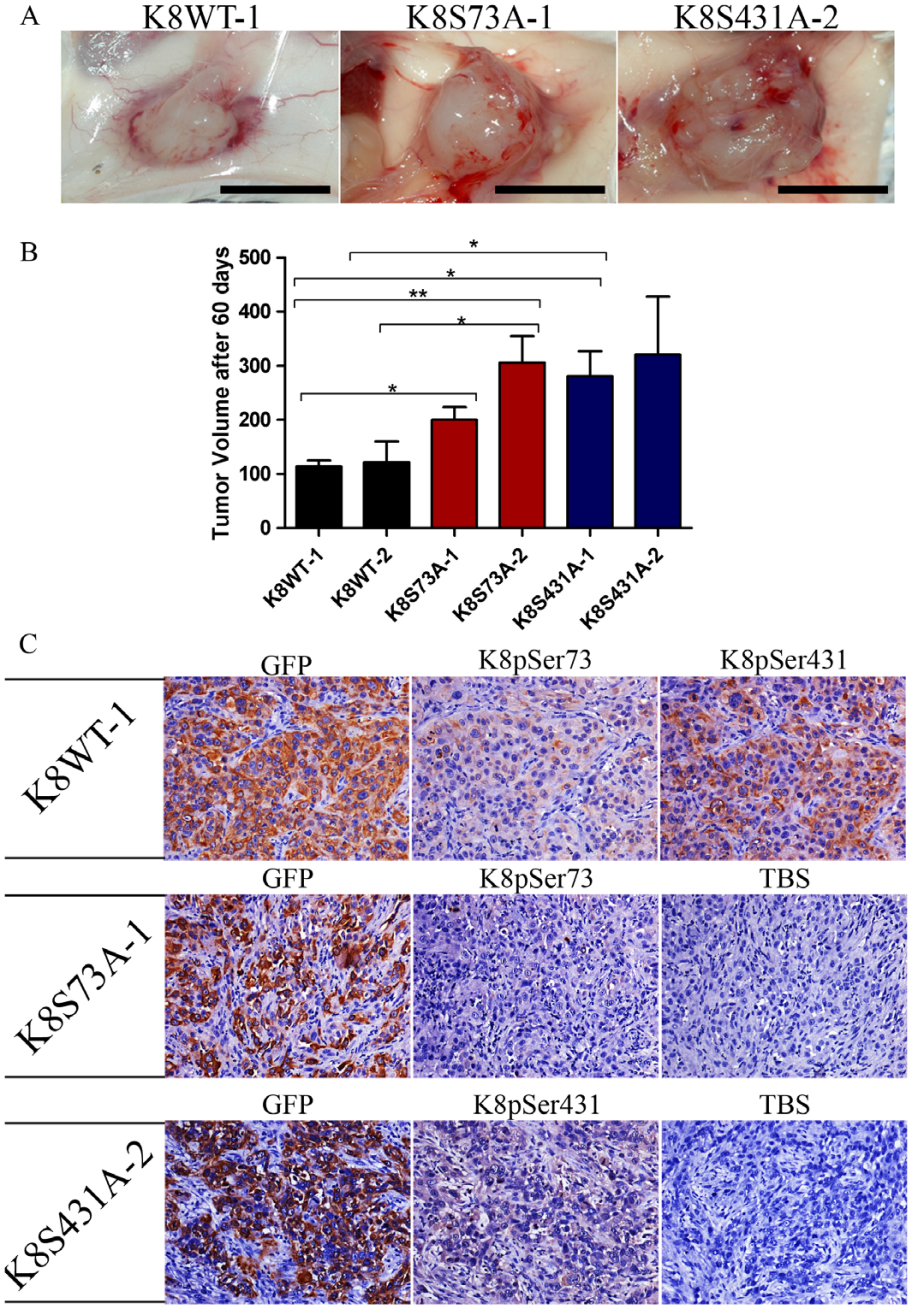
Loss of K8 phosphorylation resulted in increased tumorigenicity of AW13516 cells. (**A**) 10^3^ cells from K8 wild type (K8WT-1 and -2), K8 Ser73Ala phospho-mutant (K8S73A-1 and -2) and K8 Ser431Ala phospho-mutant (K8S431A-1 and -2) clones were injected subcutaneously into 6 different NOD-SCID mice and tumor volume measured as described. Representative images of SCID mice bearing tumors of K8WT-1, K8S73A-1 and K8S431A-2 clone 60 days after the injection. Scale bar: 10 mm (**B**) *Bars,* mean value and SD of tumor volume of K8 wild type (K8WT-1 and -2), K8-Ser73Ala (K8S73A-1 and -2) and K8-Ser431Ala (K8S431A-1 and -2) mutant after 60 days is plotted. Statistical analysis was done by Student's *t* test. * *P*<0.05; **P<0.01. (**C**) Representative images of immunohistochemical staining (with antibodies against GFP, K8-phospho-Ser73 and K8-phospho-Ser431 as indicated) of tumor tissues obtained from NOD-SCID mice injected with K8WT-1, K8S73A-1 or K8S431A-2 clones. TBS was used as a control (Magnification: 200X). Note that K8S73A-1 and K8S431A-2 clone showed loss of K8 phosphorylation at Ser73 and Ser431 respectively.

K8 and K18 are often aberrantly expressed in OSCC and their expression correlates with invasion and poor prognosis [10], [28]. K8 and K18 have been shown to promote tumorigenicity and migration in carcinoma cells including cell lines derived from OSCC [12], [14], [29]. In order to find out the role of K8 dephosphorylation on tumorigenic potential of OSCC cells, K8-Ser73Ala mutant (K8S73A-1 and -2), K8- Ser431Ala mutant (K8S431A-1 and -2) and K8 wild type (K8WT-1 and -2) clones were injected sub-cutaneously in NOD-SCID mice. In mice injected with K8 phospho-mutant clones the volume of tumor formation was significantly higher as compared to mice injected with K8 wild type clones (Figure 5A, B). To confirm the loss of K8 phosphorylation, these tumors were dissected and further analyzed for phosphorylation of Ser73 and Ser431 residue of K8 using immunohistochemistry. The tumors obtained from the K8 wild type clones showed staining of GFP and phosphorylated Ser73 and Ser431 residues of K8. The tumors developed from the K8-Ser73Ala and K8-Ser431Ala phospho-mutants did not show staining for phosphorylated K8 with respective antibodies (Figure 5C). In summary this data suggests that the loss of K8 Ser73 and Ser431 phosphorylation leads to increase in tumorigenicity of the OSCC cells.

### Loss of K8 phosphorylation in human OSCC tissues

**Figure 6:**
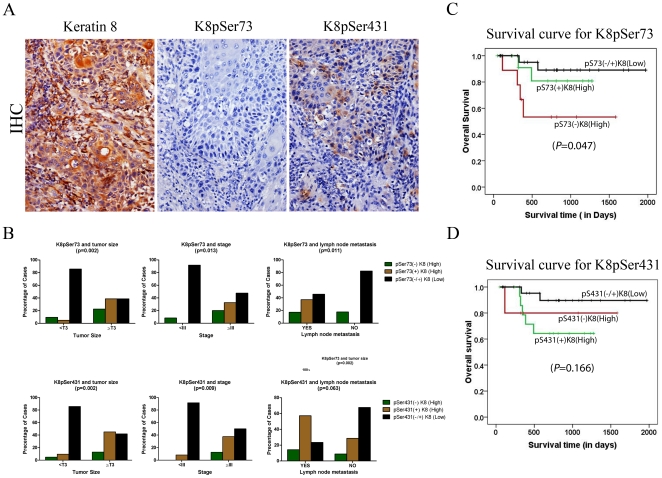
IHC analysis of K8 phosphorylation in human OSCC. (**A**) Levels of K8 phosphorylation at Ser73 and Ser431 in K8 positive paraffin embedded section of human OSCC samples were determined by immunohistochemical (IHC) analysis. Representative images of IHC staining with site specific phospho-antibodies against K8Ser73, K8Ser431 and total K8. (Magnification: 200X). (**B**) Histograms showing correlations of K8 dephosphorylation at Ser73 and Ser431 with clinico-pathological parameters such as tumor size, lymph-node metastasis and tumor stage of human OSCC patients. (**C and D**) Kaplan–Meier analysis of overall survival in OSCC patients and loss of K8 phosphorylation at Ser73 or Ser 431 residue (for K8 Ser73 phosphorylation * *P = *0.047).

In order to confirm the results of in vitro study, the levels of phosphorylated K8 were analyzed in human OSCC. A total of 52 human OSCC samples were analyzed for levels and localization of phosphorylated K8 (Ser73 or Ser431) using semi-quantitative IHC analysis (supplementary material; Figure S1). None of the non-malignant samples which were used as controls showed positive staining of K8 (supplementary material; Figure S2). All the tumor samples used in this study were K8 positive (supplementary material; Figure S2). 46% of these OSCC samples showed negative staining with phospho-K8Ser73 antibody while 58% of OSCC demonstrated negative staining with phospho-K8Ser431 antibody (Figure 6A). Thus many of human OSCC samples showed loss of K8 phosphorylation at both Ser73 and Ser431.

### Correlation of K8 dephosphorylation with clinico-pathological parameters of the patients

To evaluate the clinical significance of K8 dephosphorylation in OSCC, we investigated the correlation between levels of K8 phosphorylation at Ser73 and Ser431 residue with clinico-pathological parameters of the patients using Chi Square test. Loss of K8 phosphorylation at residue Ser73 of samples showing high K8 expression significantly correlated with tumor size (*P* = 0.002), lymph node metastasis (*P* = 0.011) and stage (*P* = 0.013) (Table 1: Figure 6B). K8 dephosphorylation at residue Ser431 of samples showing high K8 expression demonstrated statistically significant correlation with tumor size (*P* = 0.002), and stage (*P* = 0.009) (Table 1; Figure 6B). K8 dephosphorylation at residue Ser431 also showed border line significance with lymph node metastasis (Table 1; Figure 6B). Status of K8 phosphorylation did not show any correlation with other parameters. Kaplan–Meier survival analysis was conducted to establish correlation between K8 dephosphorylation and patient survival. Overall dephosphorylation of K8 at residue Ser73 showed negative correlation with patient survival (Figure 6C; *P* = 0.047), while phosphorylation at Ser431 did not correlate with patient survival (Figure 6D). Thus, our IHC analysis of tumor tissues indicated the correlation of K8 dephosphorylation with bad prognosis in OSCC patients.

**Table 1:** Correlations of K8 dephosphorylation (Ser73 and Ser431) with clinico-pathological parameters of the OSCC patients (*n* = 52).

Clinico-pathological parameter		n	Keratin 8 (K8) and pS73				Keratin 8 (K8) and pS431			
			K8(High) pS73(−) (*n* = 9)	K8(High) pS73(+) (*n* = 13)	K8(Low) pS73(−/+) (*n* = 30)	*P*-value	K8(High) pS431(−) (*n* = 5)	K8(High) pS431(+) (*n* = 16)	K8(Low) pS431(−/+) (*n* = 31)	*P*-value
**Age (Years)**	**<50**	30	4	8	18	0.673	3	9	18	0.987
	**≥50**	22	5	5	12		2	7	13	
**Sex**	**Male**	40	6	12	22	0.289	2	14	23	0.378
	**Female**	12	3	1	8		2	2	8	
**Thickness**	**<2 cm**	40	8	10	22	0.624	4	13	23	0.850
	**≥2 cm**	12	1	3	8		1	3	8	
**Stages**	**I/II**	12	1	0	11	**0.013 ^#^**	0	1	11	**0.009 ^#^**
	**III/IV**	40	8	13	19		5	15	20	
**Tumor Size**	**T1/T2**	21	2	1	18	**0.002 ^#^**	1	2	18	**0.002 ^#^**
	**T3/T4**	31	7	12	12		4	14	13	
**Lymph node metastasis**	**YES**	35	6	13	16	**0.011** *	4	14	17	0.063
	**NO**	17	3	0	14		1	2	14	
**Differentiation**	**Poor**	17	3	3	11	0.683	1	5	11	0.782
	**Moderate**	35	6	10	19		4	11	20	

*- Pearson Chi-Square, ^#^ - Spearman Correlation (Ordinal by Ordinal).

In summary, overexpression of K8 Ser73Ala and Ser431Ala mutants in K8-knockdown OSCC cells resulted in significantly increased cell migration and tumorigenicity. Furthermore, our in vitro results correlated well with IHC analysis of OSCC samples. Dephosphorylation of K8 (both at Ser73 and Ser431) was observed in many of the tumor samples. Loss of K8 phosphorylation (both at Ser73 and Ser431) correlated with tumor size, lymph node metastasis and stage. Moreover, dephosphorylation of K8 at residue Ser73 significantly correlated with poor patient survival.

## Discussion

IF form an important tissue specific compartment of cellular cytoskeleton [5]. Alterations in cellular IF expression occur as a key feature during the progression/transition from the benign to the malignant phenotype in several tumor types including OSCC [29], [30]. Although previous reports have highlighted importance of K8 and K18 expression in prognosis and progression of SCC [11], [14], [31], significance of their posttranslational modifications such as phosphorylation is not been investigated yet. In the present study, we have investigated the role of K8 phosphorylation in tumor cell migration and progression of OSCC and further tried to understand the correlation of levels of K8 phosphorylation in human OSCC tissue samples with clinico-pathological parameters of the patients.

We have used an OSCC derived cell line AW13516 as model system in this study [32]. These cells express high amount of K8 and K18 and form proper filaments. Previously, we have knockdown K8 levels using shRNA specifically targeting K8 in these cells [14]. Here, we have replaced endogenous wild type K8 with phospho-mutants, Ser73Ala and Ser431Ala of K8 by transfecting respective shRNA resistant K8-phospho mutant constructs (Figure 1 and 2). K8 knockdown background nullified the effect of phosphorylation of endogenous K8 and allowed us to compare the phenotype of wild type with phospho-mutants of K8. The stable clones expressing K8 phospho-mutants, Ser73Ala and Ser431Ala showed substantial reduction in phosphorylation of respective sites (Figure 3A, B). These phospho-mutant clones did not show significant difference in K8 and K18 filament formation compared to wild type K8 expressing OSCC cells (Figure 3C). Keratin phosphorylation is a highly dynamic process that plays an important role in regulating keratin filament organization as established from studies involving cultured cells and transgenic mouse models [16]. It appears that loss of phosphorylation at these sites may affect the dynamics of their filament assembly rather than their formation in these cells.

Dynamics of K8 and K18 filament assembly is controlled by their post translational modifications mostly by phosphorylation under various patho-physiological conditions [16], [17]. These changes in keratin filament assembly appear to be one of the determinants of the altered migratory behaviour of the cell [27]. In the present study, K8 phospho-mutants both Ser73Ala and Ser431Ala showed significant increase in cell migratory ability compared to K8-wild type clone. Results of our fluorescence microscopy analysis showed that, cells showing high expression of K8-phospho-mutants appeared on the edge of the colonies. This indicated possible increase in their migratory ability (Figure 4A). We confirmed the increase in cell migration using wound healing assay (Figure 4B). Prior studies have shown that K8 and K18 regulate cell migration in various cell types including OSCC [14], [33], [34]. Mizuuchi et al. have demonstrated correlation of PRL-3 dependent K8 dephosphorylation at Ser73 and Ser431 with increased cell motility in colorectal cancer derived-cells. They have also demonstrated the localization of K8 at cellular lamellipodias and ruffles in vivo [23]. Our data are in agreement with their findings and provide direct evidence for regulation of cell motility by K8 phosphorylation in carcinomas.

Progression of tumor cells is dependent on their migratory ability [35]. Our results of in vivo tumorigenicity assay demonstrated that loss of K8-phosphorylation (both Ser73 and Ser431) led to significant increase in tumor volume compared to K8 wild type expressing cells (Figure 5). In our previous study, we have shown reduction in tumorigenicity in response to K8 knockdown in OSCC cells [14]. We have also demonstrated that K8 and K18 filament formation promotes neoplastic transformation in oral epithelia derived cells [12]. These results together suggest that loss of K8 phosphorylation at Ser73 and Ser431 enhances tumor growth of the OSCC cells in vivo.

Next, to understand the prognostic significance of K8 dephosphorylation in human OSCC, we studied correlation of K8 dephosphorylation with clinico-pathological parameters of the patients. Here we observed loss of K8 phosphorylation at both Ser73 and Ser431 in K8 positive OSCC samples (Figure 6A; Table 1). Loss of K8 Ser73 or Ser431 phosphorylation showed statistically significant correlation with tumor size (Table 1; Figure 6B). Larger tumor size is associated with an increased risk of local recurrence, increased lymph node metastasis and poor patient survival in OSCC [36]. Loss of K8 phosphorylation also significantly correlated with tumor stage (Table 1; Figure 6B). A large number of studies have demonstrated that disease staging has a crucial influence on the outcome [37], [38]. Furthermore, dephosphorylation at Ser73 of K8 showed statistically significant correlation with lymph node metastasis while Ser431 dephosphorylation demonstrated border line significance with lymph node metastasis (Table 1; Figure 6B). Lymph node metastasis is widely accepted as one of the major prognostic factors and is associated with a decrease in overall survival and higher recurrence rates in patients with OSCC [39], [40], [41], [42]. These results are consistent with previous data on colorectal tumor tissue, which demonstrated loss of K8 phosphorylation at Ser73 and Ser431 residues, at the invasive front and in the liver metastases [23]. Moreover, loss of K8 phosphorylation at Ser73 also significantly correlated with poor survival of OSCC patients (Figure 6C). We did not observe significant correlation of K8 Ser431 dephosphorylation with patient survival probably because of smaller sample size. Thus our results indicate possible prognostic value of K8 dephosphorylation in OSCC. A study with larger sample size is required to unequivocally prove the prognostic significance of K8 dephosphorylation in OSCC.

Prior studies suggest that keratin phosphorylation is regulated in vivo by many kinases and phosphatases during various patho-physiological conditions [17]. Previously, Tao et al, have identified a phosphatase, PP2A which dephosphorylates K8 specifically at residue Ser431 under hyposmotic condition in cell culture system. Interestingly, PP2A regulates K8 phosphorylation in colonic cancer derived cell line and not in their normal counterpart [24]. Recently, Mizuuchi et al. have shown that, inhibition of PRL-3 (which directly interacts with K8) leads to increase in K8 phosphorylation at Ser73 and Ser431 along with significant reduction in cell migration in colorectal cells [23]. These observations also correlated with higher PRL-3 expression and reduced K8 phosphorylation at both the sites in tumor tissue samples [23]. Importantly, Hassan et al. have demonstrated higher PRL-3 expression in OSCC and dysplasia compared to normal oral tissues [43]. These findings together suggest that loss of K8 phosphorylation may be due to higher PRL-3 activity in OSCC samples. Further studies are necessary to establish the role of phosphatases in K8 phosphorylation dependent progression of carcinomas.

In summary, our results clearly suggest that loss of K8 phosphorylation leads to increased cell migration and tumorigenicity in OSCC cells. In addition, K8 dephosphorylation was also observed in human OSCC samples which correlated with tumor size, stage and lymph node metastasis. Furthermore, loss of K8 Ser73 phosphorylation significantly correlated with poor patient survival indicating its significance in prognosis. Our results support Mizuuchi et al's hypothesis that phosphorylation dependent reorganization of keratins accelerate cell motility and metastatic potential of carcinomas [23]. However, the exact role K8 dephosphorylation in cell motility and tumor progression is yet to be elucidated. Thus, the present study provides important insights into the understanding the role of keratin phosphorylation during tumor progression and K8 dephosphorylation shows a good promise to be used as prognostic marker. Moreover, intervention strategies to inhibit phosphatase mediated K8 dephosphorylation in OSCC might lead to the development of novel therapeutic targets for invasive and metastatic carcinomas.

## Materials and Methods

### Ethics statement

This study was approved by the “Tata Memorial Hospital Human Ethics Committee”. The written informed consent was obtained from the patients as well as healthy individuals. All protocols for animal studies were reviewed and approved by the “Institutional Animal Ethics Committee (IAEC)” constituted under the guidelines of the “Committee for the Purpose of Control and Supervision of Experiments on Animals (CPCSEA)”, Government of India (Approval ID: 15/2007).

### Cell lines, plasmids and site directed mutagenesis

The cell line AW13516 derived from the SCC of tongue [32] and HEK-293 (ATCC) were cultured in IMDM and DMEM (Gibco), respectively, supplemented with 10% fetal calf serum (FCS; Hyclone) and antibiotics, at 37°C and under 5% CO_2_ atmosphere [14].

To generate shRNA resistant K8-GFP wild type (K8-GFP-WT-R), K8-Ser73 mutant (K8-GFP-S73A-R) and K8-Ser431 mutant (K8-GFP-S431A-R), Quick change Site directed mutagenesis kit (Stratagene) was used (K8-phospho-mutant constructs are a kind gift of Dr. Normand Marceau, Canada) [44]. Primer 5′-GGAGGCATCACCGCTGTGACGGTCAACCAGAGC-3′ was synthesized as per manufacturer's instructions, containing 2 silent mutations in the shRNAK8.2 binding site [14]. The resulted mutations were verified by direct DNA sequencing.

### Transfection and selection of stable clones

To investigate the effect of K8 phosphorylation on cell migration and tumorigenicity, the shRNA resistant K8-GFP wild type (K8-WT-GFP-R), K8-Ser73 mutant (K8-S73A-GFP-R) and K8-Ser431 mutant (K8-S431A-GFP-R) were overexpressed in K8-knockdown clone shRNAK8.2-C1. The stable K8-knockdown clones were generated as described previously [14]. Briefly, 2 µg of shRNAK8 construct (Target site: given in Figure 1) or the empty vector control were transfected in AW13516 cells, by liposome-based FuGENE HD transfection reagent (according to manufacturer's protocol; Roche). The stable K8-knockdown clones were selected in medium containing 0.5 µg/ml puromycin (Sigma). Similarly, to generate stable K8 wild type and phospho-mutants overexpressed clones, 2 µg of plasmid DNAs were transfected into the K8-knockdown cells. The cells were selected (second selection) in 1000 µg/ml G418 sulphate containing medium. Single cell clones were isolated, expanded and screened for the overexpression of K8 wild type and phospho-mutants using laser confocal microscopy, RT-PCR and western blot analysis.

### Western blot analysis

Whole cell lysates were prepared in SDS lysis buffer (2% SDS, 50 mM Tris-HCl pH 6.8, 0.1% BME and 10% glycerol) with protease inhibitors cocktail (Calbiochem). Equal amount of protein was loaded and run on SDS-PAGE. The gels were transferred on PVDF membrane (Hybond Amersham) and probed with the primary antibody followed by secondary antibody conjugated with HRP (Amersham). The primary antibodies used were as follows: K8 (Sigma; working dilution 1∶8000), K18 (Sigma; dilution 1∶8000), GFP (Clonetech; dilution 1∶8000), Keratin8-pSer73 (LifeSpan Biosciences; dilution 1∶1000), Keratin8-pSer431 (Abcam; dilution 1∶1000) and β-actin (Sigma; dilution 1∶8000). The signals were detected using ECLplus detection system (Amersham) according to manufacturer's protocol.

### Immunofluorescence and laser confocal microscopy

To detect the localization of proteins and filament organization of keratins in cells, immunofluorescence assay was performed as described previously [45]. Briefly, the cells were grown on glass cover slips for 48 hrs and were fixed with 100% chilled methanol. After fixation, coverslips were washed with 1X PBS and then permeabilized using 0.3% Triton X-100 in methanol. After permeabilization, the cells were blocked with 5% BSA for 1 hr. The cells were then layered with 50 µl of 1∶200 diluted K18 antibody and incubated for 1 hr. The coverslips were washed with 1XPBS followed by incubation with 50 µl of 1∶200 diluted Alexa-Fluor-568-conjugated anti-mouse-IgG secondary antibody (Molecular probes) for 1 hr. Coverslips were then washed and mounted using antiquenching agent and confocal images were obtained using a LSM 510 Meta Carl Zeiss Confocal system.

### RNA isolation and reverse transcriptase-polymerase chain reaction (RT-PCR)

RNA was isolated by TRI reagent (Sigma) and RT-PCR was conducted using RevertAid™ First Strand cDNA synthesis Kit (Fermentas) according to manufacturer's protocol. The following primers were used, Keratin 8-forward 5′-AGATGAACCGGAACATCAGC-3′, reverse 5′-AGCAGCTTCCTGTAGGT- 3′ and *GAPDH* forward 5′-GAAGGTGAAGGTCGGAGTC-3′ reverse 5′-GAAGATGGTGATGGGATTTC-3′. PCR conditions were as follows: denaturation at 94°C for 30 s annealing at 56°C and 58°C for Keratin 8 and *GAPDH* respectively for 30 s and extension at 72°C for 60 s. PCR products were run on agarose gel electrophoresis to compare RNA levels.

### Fluorescence microscopy analysis and wound-healing assay for cell motility

To check the effect of loss of K8 phosphorylation on cell motility, differential distribution of K8 phospho-mutants and wild type expressing cells was analyzed using inverted fluorescence microscopy. The cells were seeded in 35 mm tissue culture plates in selection medium and allowed to grow for 48 hrs. The GFP expression was analyzed and images of 5 different fields of the clones were taken using an AxioCam MRm camera. Wound-healing assay was performed as described previously. Briefly, the cells were grown in 35 mm plates to 95% confluency and were replaced with fresh medium containing 20 µg/ml mitomycin C to inhibit cell proliferation. After 3 hr incubation, medium was discarded and wounds were scratched with the help of sterile 2 µl pipette tip. The cells were fed with fresh medium and observed by time lapse microscopy, and images were taken every 10′ for 20 hr. Migration was measured using Axiovision software version 4.5 (Zeiss).

### Tumor formation in NOD-SCID mice

To test the tumorigenicity of the cells the NOD-SCID mice (6–8 weeks old) were used. AW13516 clones (both K8 wild type and phospho-mutants overexpressed cells) were suspended in plane IMDM without serum, 3×10^6^ cells were injected sub-cutaneously in the dorsal flank of 6–8 weeks old NOD-SCID mice. Six mice were injected for each clone and observed for 60 days for tumor formation. The ellipsoid volume formula 1/2 x L x W x H was used to calculate the tumor volume [14].

### Surgical specimens and clinico-pathological records of the patients

Tumor tissues were collected from the Tata Memorial Hospital (TMH), Parel, Mumbai, India. A total of 52 K8 positive primary oral SCC (34 SCC of tongue and 18 SCC of buccal mucosa) were selected for IHC analysis. Five punch biopsy samples of patients suffering from inflammatory fibrous hyperplasia of oral cavity (2 BM and 3 Gingival) were obtained from Nair Dental College, Mumbai, India. The patients had no history of malignancy. These tissues were used as controls. Clinico-pathological information like tumor site, size, histological grade, perineural invasion, perineural extension, cut margins, bone and lymph node involvement, loco-regional recurrence, metastasis and survival was collected from the clinical and pathology records of Tata memorial Hospital (supplementary material; Table S1).

### Histology and immunohistochemistry (IHC)

Tissue samples were fixed in 10% formalin buffer and five micron sections were cut from paraffin embedded blocks. Sections were stained with hematoxylin and eosin (H&E) for histological diagnosis. Immunohistochemical staining was performed using Elite ABC Kit (Vector laboratories; USA) as described earlier [45]. The primary antibodies used were against K8 (dilution 1∶200), phosphorylated Ser73 of K8 (dilution 1∶100) and phosphorylated Ser431 of K8 (dilution 1∶200). The negative control was kept with tris-buffered saline instead of primary antibody. Specimens were divided into following categories 1. For K8, High (homogenous positive tumor in more than 50% of cells or/and high membranous and cytoplasmic staining in more than 50% of cells; ++/+++) and low (20–50% positive tumor cells; +) 2. For phosphorylated K8-Ser73 and Ser431, positive (homogenous positive tumor in more than 20% of cells or/and high membranous and cytoplasmic staining in more than 20% of cells; +/++/+++) and negative (less than 20% positive cells or no evidence of staining; −). Results of IHC were independently assessed by two observers.

### Statistical analysis

To assess correlations between clinico-pathological parameters and results of IHC, the *Chi Square* test was used. Univariant analysis was performed using the Kaplan-Meier method and statistical significance between survival curves was assessed by the log rank tests. The data were analyzed with the Statistical Package, SPSS 16.0 for Windows (SPSS Inc., Chicago, IL, USA). Two groups of data were statistically analyzed by t *test* using Graphpad Prism5 software. A *P* value less than 0.05 was considered statistically significant.

## Supporting Information

Figure S1 **IHC analysis of K8 expression in malignant and non-malignant human oral tissues.** Representative images of H&E and IHC staining using antibody against K8 on paraffin embedded sections of human oral tumors and non-malignant tissues. Note that non-malignant oral tissues showed negative staining while malignant tissues showed positive staining of K8.
(TIF)

Figure S2 **IHC staining of phosphorylated K8 in human OSCC.** Representative images of H&E staining along with positive IHC staining with antibodies specific to phosphorylated Ser73 and Ser431 of K8 on paraffin embedded sections of human OSCC.
(TIF)

Table S1 **Clinico-pathological parameters of OSCC patients (** ***n*** **= 52).**
(DOC)
