# Supplementary figures and images for: Loss of Keratin 8 Phosphorylation Leads to Increased Tumor Progression and Correlates with Clinico-Pathological Parameters of OSCC Patients
Source: PLoS One. 2011 Nov 17;6(11):e27767. doi: 10.1371/journal.pone.0027767 (PMC3219681; doi:10.1371/journal.pone.0027767)

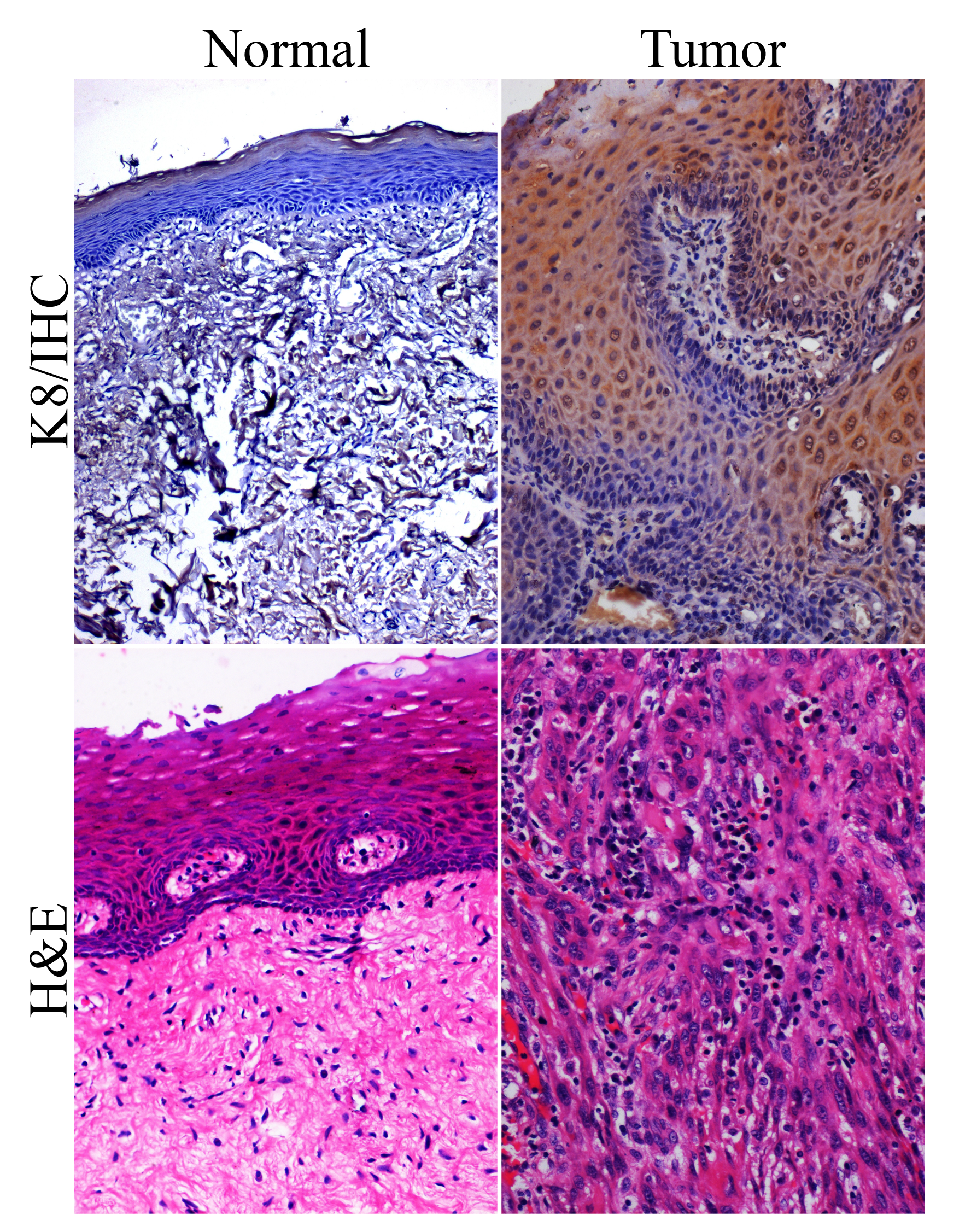

Supplement: Figure S1 — IHC analysis of K8 expression in malignant and non-malignant human oral tissues. Representative images of H&E and IHC staining using antibody against K8 on paraffin embedded sections of human oral tumors and non-malignant tissues. Note that non-malignant oral tissues showed negative staining while malignant tissues showed positive staining of K8. (TIF) [file pone.0027767.s001.tif]

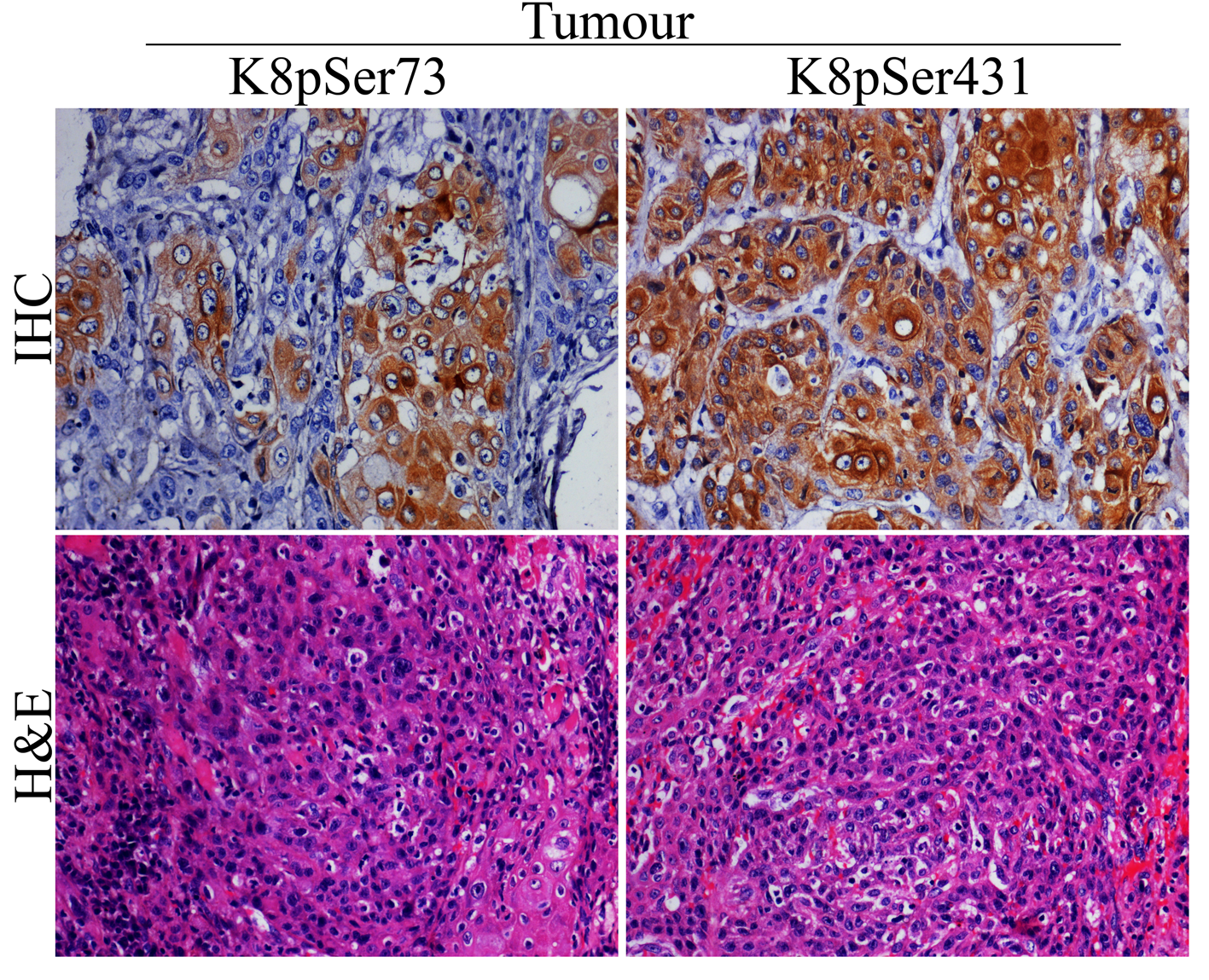

Supplement: Figure S2 — IHC staining of phosphorylated K8 in human OSCC. Representative images of H&E staining along with positive IHC staining with antibodies specific to phosphorylated Ser73 and Ser431 of K8 on paraffin embedded sections of human OSCC. (TIF) [file pone.0027767.s002.tif]
