# Supplementary material for: Loss of Keratin 8 Phosphorylation Leads to Increased Tumor Progression and Correlates with Clinico-Pathological Parameters of OSCC Patients
Source: PLoS One. 2011 Nov 17;6(11):e27767. doi: 10.1371/journal.pone.0027767 (PMC3219681; doi:10.1371/journal.pone.0027767)
Supplement: Table S1 — Clinico-pathological parameters of OSCC patients ( n = 52). (DOC) [file pone.0027767.s003.doc]

| **Clinico-pathological parameter** | | **n (%)** |
| --- | --- | --- |
| Age (Years) | <50 | 30 (57.69) |
|  | ≥50 | 22 (42.31) |
| Sex | Male | 40 (76.92) |
|  | Female | 12 (23.08) |
| Location | Tongue | 34 (65.38) |
|  | BM | 18 (34.62) |
| Thickness | <2cm | 40 (76.92) |
|  | ≥2cm | 12 (23.08) |
| Stages | I/II | 12 (23.08) |
|  | III/IV | 40 (76.92) |
| Tumor  size | T1/T2 | 21 (40.38) |
|  | T3/T4 | 31 (59.62) |
| Lymph node metastasis | YES | 35 (67.31) |
|  | NO | 17 (32.69) |
| Differentiation | Poor | 17 (32.69) |
|  | Moderate | 35 (67.31) |
| Bone involvement | Positive | 6 (11.54) |
|  | Negative | 30 (57.69) |
| Perineural invasion | Yes | 10 (19.23) |
|  | No | 33 (63.46) |
| Perineural extension | Yes | 25 (48.08) |
|  | No | 20 (38.46) |
| Resection margin | Free | 47 (90.38) |
|  | Closed | 4 (7.69) |
|  | Invasion | 1 (1.92) |
| Recurrence | Yes | 14 (26.92) |
|  | No | 34 (65.38) |
| Skin involvement | Yes | 2 (3.85) |
|  | No | 26 (50) |

Table S1: Clinico-pathological parameters of OSCC patients.
